# Gait Kinematic Analysis in Water Using Wearable Inertial Magnetic Sensors

**DOI:** 10.1371/journal.pone.0138105

**Published:** 2015-09-14

**Authors:** Silvia Fantozzi, Andrea Giovanardi, Davide Borra, Giorgio Gatta

**Affiliations:** 1 Department of Electrical, Electronic, and Information Engineering «Guglielmo Marconi», University of Bologna, Bologna, Italy; 2 School of Pharmacy, Biotechnology and Sport Science, University of Bologna, Bologna, Italy; 3 Department for Life Quality Studies, University of Bologna, Bologna, Italy; Faculté de médecine de Nantes, FRANCE

## Abstract

Walking is one of the fundamental motor tasks executed during aquatic therapy. Previous kinematics analyses conducted using waterproofed video cameras were limited to the sagittal plane and to only one or two consecutive steps. Furthermore, the set-up and post-processing are time-consuming and thus do not allow a prompt assessment of the correct execution of the movements during the aquatic session therapy. The aim of the present study was to estimate the 3D joint kinematics of the lower limbs and thorax-pelvis joints in sagittal and frontal planes during underwater walking using wearable inertial and magnetic sensors. Eleven healthy adults were measured during walking both in shallow water and in dry-land conditions. Eight wearable inertial and magnetic sensors were inserted in waterproofed boxes and fixed to the body segments by means of elastic modular bands. A validated protocol (Outwalk) was used. Gait cycles were automatically segmented and selected if relevant intraclass correlation coefficients values were higher than 0.75. A total of 704 gait cycles for the lower limb joints were normalized in time and averaged to obtain the mean cycle of each joint, among participants. The mean speed in water was 40% lower than that of the dry-land condition. Longer stride duration and shorter stride distance were found in the underwater walking. In the sagittal plane, the knee was more flexed (≈ 23°) and the ankle more dorsiflexed (≈ 9°) at heel strike, and the hip was more flexed at toe-off (≈ 13°) in water than on land. On the frontal plane in the underwater walking, smoother joint angle patterns were observed for thorax-pelvis and hip, and ankle was more inversed at toe-off (≈ 7°) and showed a more inversed mean value (≈ 7°). The results were mainly explained by the effect of the speed in the water as supported by the linear mixed models analysis performed. Thus, it seemed that the combination of speed and environment triggered modifications in the joint angles in underwater gait more than these two factors considered separately. The inertial and magnetic sensors, by means of fast set-up and data analysis, can supply an immediate gait analysis report to the therapist during the aquatic therapy session.

## Introduction

The aquatic environment is a useful tool for rehabilitative motor activities of patients with compromised, damaged, or impaired posture or locomotion [1]. The buoyancy of water reduces the effective gravitational load on joints depending on the immersion depth [2]. The drag force, providing resistance to movements, can be varied modifying the execution speed of motor tasks [3]. Furthermore, the pressure exerted by water involves an increased proprioception that allows higher safety and less stress for patients [4]. The lower risk of fall enables patients to execute the required motor task with a lower muscular tension [5]. Finally, thanks to the fast thermal convection of water, the fluid temperature can be modified in order to produce effects on the cardiovascular, muscolo-skeletal, endocrine and neuro-vegetative systems of patients [6].

Walking is one of the most common motor tasks in water-based exercise programs and, frequently, it can be performed even when walking is not possible in dry-land (DL) condition. This advantage allows early analysis of the function and the coordination of the main chains (postural and motor) of the body. Several studies performed a gait analysis in water using waterproofed video cameras [7–13]. Kinematic, kinetic and electromyographical analyses of the lower limbs were performed and compared to those of DL condition. However, the previous studies reported kinematic analyses only for the sagittal plane and the lower limbs, without taking into account frontal and transverse planes and/or thorax-pelvis kinematics. Furthermore, the underwater video cameras (UW) analyses has several drawbacks: the limited field of view allows for analysis of only one/two consecutive steps, set-up and post-processing are time-consuming, and the accuracy is lower than DL condition due to the refraction of light and the parallax error at the water-air interface [14–16]. These drawbacks restrict aquatic analyses of physiotherapists to only a delayed UW kinematics observation and do not permit a prompt assessment of the correct execution of aquatic movements during the aquatic therapy session.

In the last few years, the use of wearable inertial and magnetic sensor units (IMMUs) for gait analysis has gained enough accuracy to be used in a rehabilitation context [17,18]. The improvements in dimensions, waterproofing, and cost of IMMUs have allowed for the use of motion analysis even in underwater environment [19]. Using IMMUs, gait analysis can be performed not only in the laboratory, but also in daily life contexts, since there is no limitation in the dimension of the acquisition field; however, limitations can arise from the wireless transmission range or data memory storage capacity. In addition, several consecutive steps can be acquired and quick set-up and post-processing enable a fast reporting that can be analyzed by physiotherapists just after the aquatic exercises is performed by patients.

The aim of the present study was to estimate the joint kinematics of the lower limbs and of the thorax-pelvis in sagittal and frontal planes during walking in water using IMMUs. Joint angle patterns of UW walking were compared with those of the same participants in DL condition.

## Materials and Methods

Bioethics Committee of the University of Bologna approved this research.

### Protocol

The Outwalk protocol [17] was chosen for estimating thorax-pelvis and lower limb kinematics using IMMUs as it entails fast set-up and comfortable calibration procedures with an accuracy comparable to that of optoelectronic systems [20].

Specifically, in the Outwalk protocol, the body is modeled as an open kinematic chain constituted by 8 rigid segments (thorax, pelvis, both thighs, both shanks, both feet) with 21 degrees of freedom. Posterior-anterior tilting, right drop-rise, and right internal-external rotation were calculated for the thorax-pelvis joint. Flexion-extension, abduction-adduction, and internal-external rotation were calculated for the hip and the knee joints. Dorsi-plantar flexion, ankle inversion-eversion, and internal-external rotation were calculated for the ankle joint. The thorax-pelvis, the hip and the ankle were considered ball-and-socket joints, while the knee was considered as a ‘loose’ double-hinge joint. Several previous studies on 3D joint kinematics of the lower limb found that the estimation of the joint angles of the transversal plane is not accurate and reliable enough when using stereophotogrammetry and IMMUs [20,21]. Thus, consistently with the aim of this study, the right internal-external rotation of the thorax-pelvis and the internal-external rotation of the hip, the knee, and the ankle were not considered.

One static calibration and two (one for each side) functional calibrations allowed an optimal definition of the anatomical reference systems. The static calibration was performed with the participant standing in an upright posture for 20 seconds, while the functional calibration consisted in the knee flexion-extension (repeated five times) up to 70° of flexion at a self-selected speed. While the first calibration established mainly the joint angle offsets of the hip and the ankle, the second calibration allowed for the orientation estimation of the mean flexion-extension axis of the knee.

For the right side the following anatomical axes definitions were used: *y* vertical pointing up, *x* anterior-posterior pointing anteriorly and *z* medio-lateral, pointing laterally. For the left side: the *y* vertical pointing down, *x* anterior-posterior pointing posteriorly and *z* medio-lateral pointing medially. The anatomical axes described allowed for the use of the *zxy* Euler convention to decompose joint angles. More details regarding the protocol can be found in Cutti et al. [17].

### Participants and Motor Tasks

Eleven healthy participants (6 males and 5 females, 27.0 ± 3.4 years, 174.2 ± 8.2 cm height, 70.2 ± 11.8 Kg mass) were measured during walking in water (1.2 m depth at a temperature of 28°C) and during walking in DL condition. Three 10 m walking barefoot trials at a self-selected comfortable speed for each condition were measured. To avoid any modification of the gait pattern in both conditions, no device, such as a metronome or a timer, was used to enforce the walking speed [22]. In the UW condition, participants executed the three trials once adapted to the water environment with no restrictions of the upper-limb movement. For that reason, a warm-up session before the execution of the three trials was required.

To avoid ferromagnetic disturbance to the IMMUs, the walking was performed at a minimal distance of 150 cm from the border of the swimming pool. The participants had no neurological or musculoskeletal pathologies and gave written informed consent to participate in this study that was approved by the Bioethics Committee of the University of Bologna.

### Set-Up

**Fig 1:**
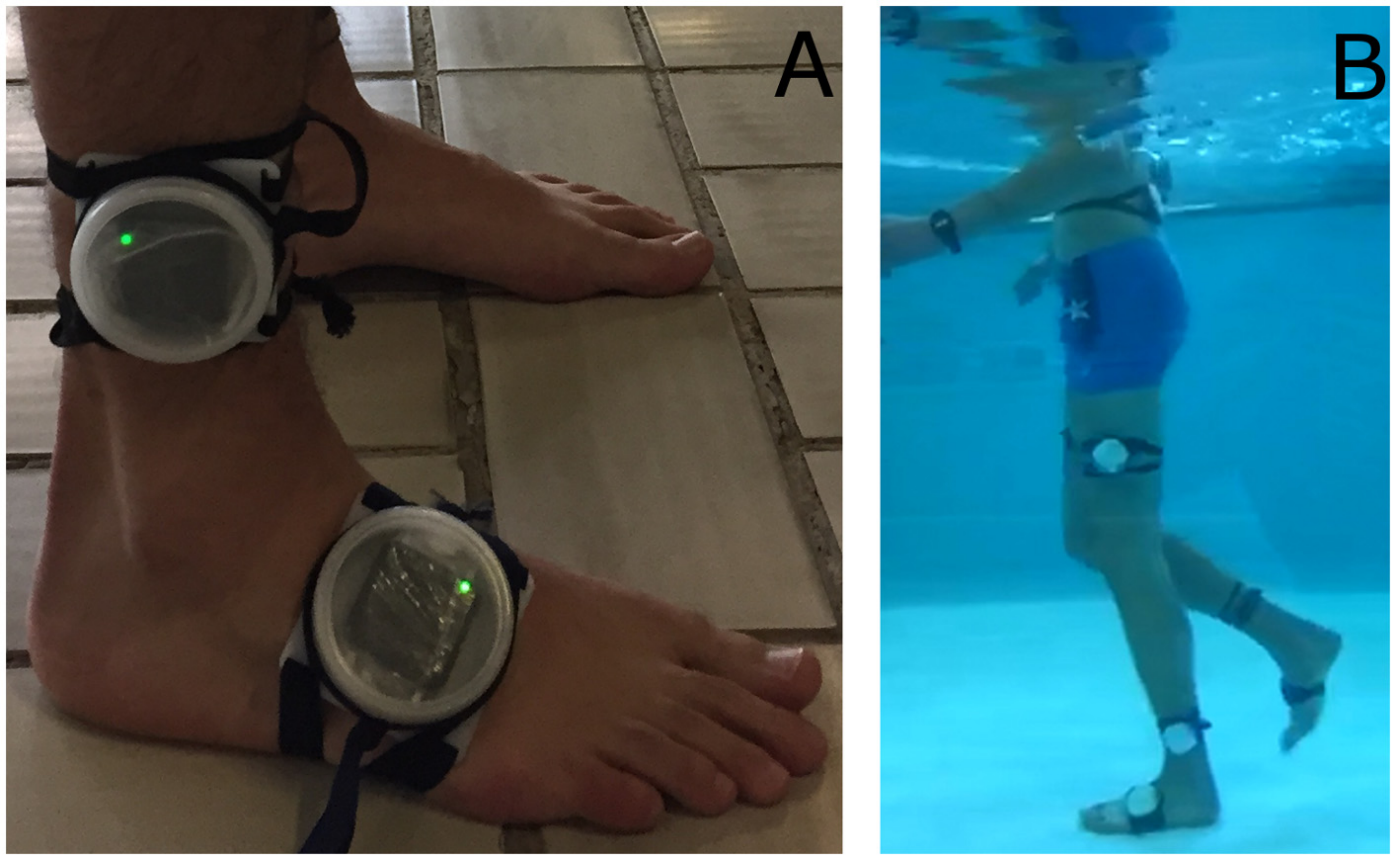
Set-up protocol. Round plastic boxes for waterproofing the sensors (A). 8 IMMUs fixed to the body segments of the participant by means of elastic bands (B).

Eight IMMUs (Opal, APDM, 128 Hz, Motion Studio software beta version 1.0.0.201310221707) were calibrated at the beginning of each acquisition session, inserted in round plastic waterproofed boxes and fixed to the body segments of the participant by means of elastic bands (Fig 1). Adhesive, tape and adhesive spray were used to ensure that the boxes remained fastened to the skin. The same operator outfitted all the participants. The following indications on positioning the IMMUs on the participant’s body were followed to ensure the maximum accuracy of the protocol and the reduction of the soft-tissue artifacts. The IMMU on the thorax was placed in the middle area between the *incisura jugularis* and *processus xiphoideus*, aligning the *x*-Opal-axis to the long axis of the sternum. The IMMU on the pelvis should be placed with the *x*-Opal-axis aligned with the left-right axes line. The IMMUs on the thighs were placed in the central-third, with the *z*-Opal-axis pointing laterally. The IMMUs on the shanks were placed slightly above the lateral malleolus, with the *z*-Opal-axis pointing perpendicular to the sagittal plane. The IMMUs on the feet were placed over the flat portion of the lateral part of the metatarsal area.

### Data Analysis

Data collection was performed using the Motion Studio Software (APDM, USA), while the data processing was performed using Matlab® language (The Mathworks^TM^, USA).

The IMMUs estimation of the orientation was computed combining raw data from the gyroscopes, accelerometers and magnetometers through the Kalman fusion filter proposed by Madgwick [23]. The β gaining factor defined by Madgwick was previously tuned for the specific motor task, comparing inertial data with optoelectronic data in laboratory controlled conditions (BTS Smart-DX 7000). The optimal β was 0.312.

Anatomical systems of references were obtained applying the Outwalk protocol to the orientation matrices of the sensors. Joint matrices were computed between adjacent segments and joint angles were then estimated decomposing joint matrices. Finally, a 3 Hz 2^nd^ order Butterworth low-pass filter was applied to all joint angles.

A statistical analysis of the kinematics of the lower limb during DL and UW walking required that joint angles be segmented and time-normalized into gait cycles. Thus, for each environment, gait cycles were obtained using an automatic segmentation algorithm proposed by Aminian [24] for DL walking, that was previously adapted to UW walking. To have the same number of gait cycles for each participants and each environment, a selection procedure and a deletion procedure were implemented. The selection procedure was based on the Intraclass Correlation Coefficient (ICC) and executed for each trial. Typically, an ICC value that characterizes a good agreement between measures/cycles has a value greater than a threshold of 0.75 [25]. Since the more reliable joint angles in gait analysis are those of the sagittal plane, a gait cycle was considered for subsequent analysis only when the ICC values of hips, knees, and ankles flexion-extension angles were simultaneously greater than the threshold value.

The deletion procedure was executed to gain the same number of gait cycles for each participants and for each environment in the three walking trials. At the end of these two procedures, for each environment analyzed, all cycles collected for all participants and for all trials were merged together. Thus, the total number of gait cycles considered was 352 for each degree of freedom of each joint.

Because hip, knee and ankle joint angles were computed for both right and left side and because all the participants were healthy, joint angles were grouped into knee, hip and ankle joints without side dependence. Thus, the total number of gait cycles considered was 704 for each degree of freedom of these joints. Finally, normality bands were computed.

The following spatio-temporal variables were calculated for each gait cycle: stride, stance and swing times [s], stance and swing percentages [%], stride length [cm], number of steps, and walking speed [cm/s]. Furthermore, once a specific degree of freedom of a joint was selected, the following angular variables were calculated for each cycle and for each side: Range Of Motion (ROM) [deg], minimum [deg], maximum [deg], percentages of gait cycle at minimum and at maximum [%], and values of the angle at heel strike and at toe-off [deg]. To investigate the sensitivity of the results to the number of cycles, spatio-temporal and angular variables were estimated for three, two and one trial using an ICC value greater than 0.75.

Linear mixed models were used to identify the effects of the environment (DL/WL), of the walking speed, and their interaction on each outcome variable. The models were specified as follows:

$$y_{ij}=\beta_{0}+u_{oj}+\beta_{1}\cdot x_{1ij}+\beta_{2}\cdot x_{2ij}+\beta_{3}\cdot (x_{1ij}\cdot x_{2ij})+\varepsilon_{0ij}$$ (1)

where *u*_*oj*_ represent the random effects associated to the j^th^ subject, the subscripts ij indicate values of variables measured in the i^th^ trial clustered in the j^th^ subject, *y* represents the outcome variable, *x*_1_ represents the environment (coded with a dummy equal to 1 or 0 for the WL and DL conditions, respectively), *x*_2_ represents the walking speed, and *ε*_0_ represents the random error component. The analyses were performed using the R statistical software (version 3.0.2).

## Results

Generally, higher standard deviations of the parameters were found in the UW condition (Table 1). However, considering each single participants, the relevant standard deviation was similar to that of the DL condition.

**Table 1:** Mean and standard deviation values (n = 704 gait cycles in 11 participants) of the parameters that were found different and explained by the different effect of environment and/or of walking speed. The coefficient of the linear mixed models were reported. * referred to values with p<0.05.

		Mean ± std.dev		Coefficients of Linear mixed models					
		*Dry-land*	*Under-water*	β_0_	β_1_	β_2_	β_3_
**Spatio-temporal**	Stride duration [s]	1.1 ± 0.1	2.8 ± 0.7	*2*.43*	*2*.79*	*-0*.01*	*-0*.03*
	Stance percentage [%]	57.9 ± 2.6	59.8 ± 4.6	*0*.72*	*-0*.08*	*-0*.00*	*0*.00*
	Stride distance [cm]	161.2 ± 13.8	150.2 ± 12.1	*124*.14*	*21*.87*	*0*.25*	*-0*.18*
**Hip**	Flexion-Extension at toe-off [deg]	-2.1 ± 5.7	11.2 ± 9.1	*13*.42*	*-24*.00*	*-0*.11*	*0*.48*
	Flexion-Extension maximum [deg]	25.0 ± 3.0	32.9 ± 7.2	*22*.89*	*3*.*00*	*0*.*01*	*0*.11*
	Flexion-Extension minimum [deg]	-11.1 ± 3.9	-2.7 ± 6.6	*-3*.86*	*-14*.48*	*-0*.05*	*0*.32*
**Knee**	Flexion-Extension at heel strike [deg]	-3.5 ± 5.7	19.4 ± 7.7	*7*.65*	*-0*.*96*	*-0*.08*	*0*.29*
	Flexion-Extension maximum [deg]	56.8 ± 4.4	64.9 ± 18.8	*85*.35*	*-14*.67*	*-0*.19*	*0*.09*
	Flexion-Extension Range of Motion [deg]	64.9 ± 3.8	60.0 ± 18.0	*80*.99*	*-13*.31*	*-0*.11*	*-0*.*02*
**Ankle**	Dorsi-Plantar flexion at heel strike [deg]	-6.6 ± 5.3	2.8 ± 7.8	*4*.*27*	*-22*.17*	*-0*.07*	*0*.43*
	Dorsi-Plantar flexion Range of Motion [deg]	29.8 ± 4.3	38.4 ± 13.7	*50*.75*	*16*.65*	*-0*.14*	*-0*.36*
	Inversion-Eversion at toe-off [deg]	11.0 ± 6.2	17.5 ± 8.7	*22*.67*	*4*.*55*	*-0*.08*	*-0*.11*
	Inversion-Eversion mean [deg]	3.1 ± 2.7	10.3 ± 5.8	*7*.20*	*7*.06*	*-0*.03*	*-0*.04*

Regarding the spatio-temporal parameters, a lower walking speed was found in the UW condition (57.9 ± 14.5 cm/s) compared to the DL condition (146.7 ± 18.8 cm/s). Furthermore, a longer stride duration and a shorter stride distance were found in the UW condition (Table 1). This latter result can be explained by the different effect of the walking speed in the two conditions: an increased value of 0.25 cm of the stride distance was estimated for each one cm/s increase of walking speed in DL condition, whereas an increase of 0.07 cm was found in the UW condition (Table 1).

Thus, considering the mean walking speed value, this effect led to a distance of about 4.05 cm in UW condition.

**Fig 2:**
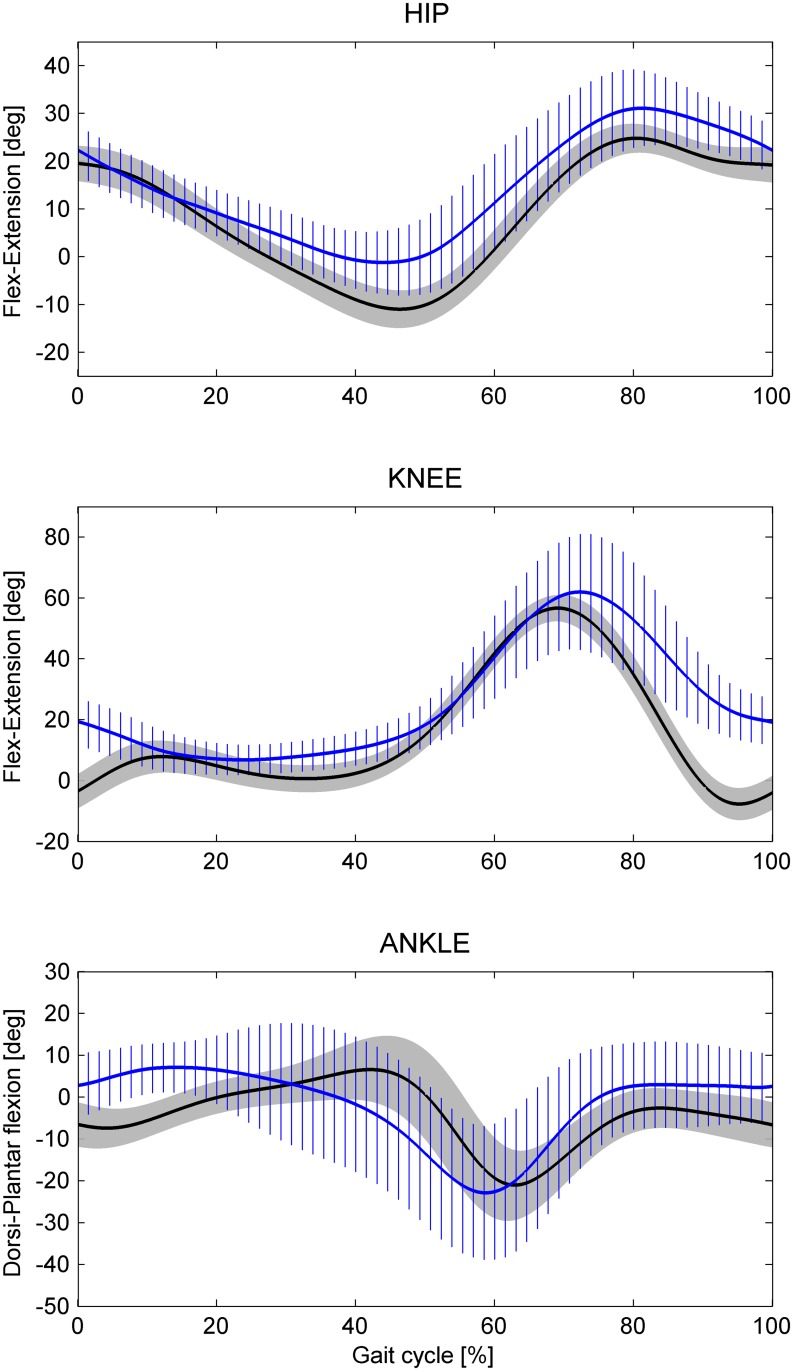
Angular kinematic patterns of the lower limb joints (hip, knee, and ankle) in the sagittal plane. Mean values plus and minus one standard deviation for all the participants for Dry-Land (black solid line and grey shaded area) and Under-Water (blue solid line and blue stripes area) conditions.

**Fig 3:**
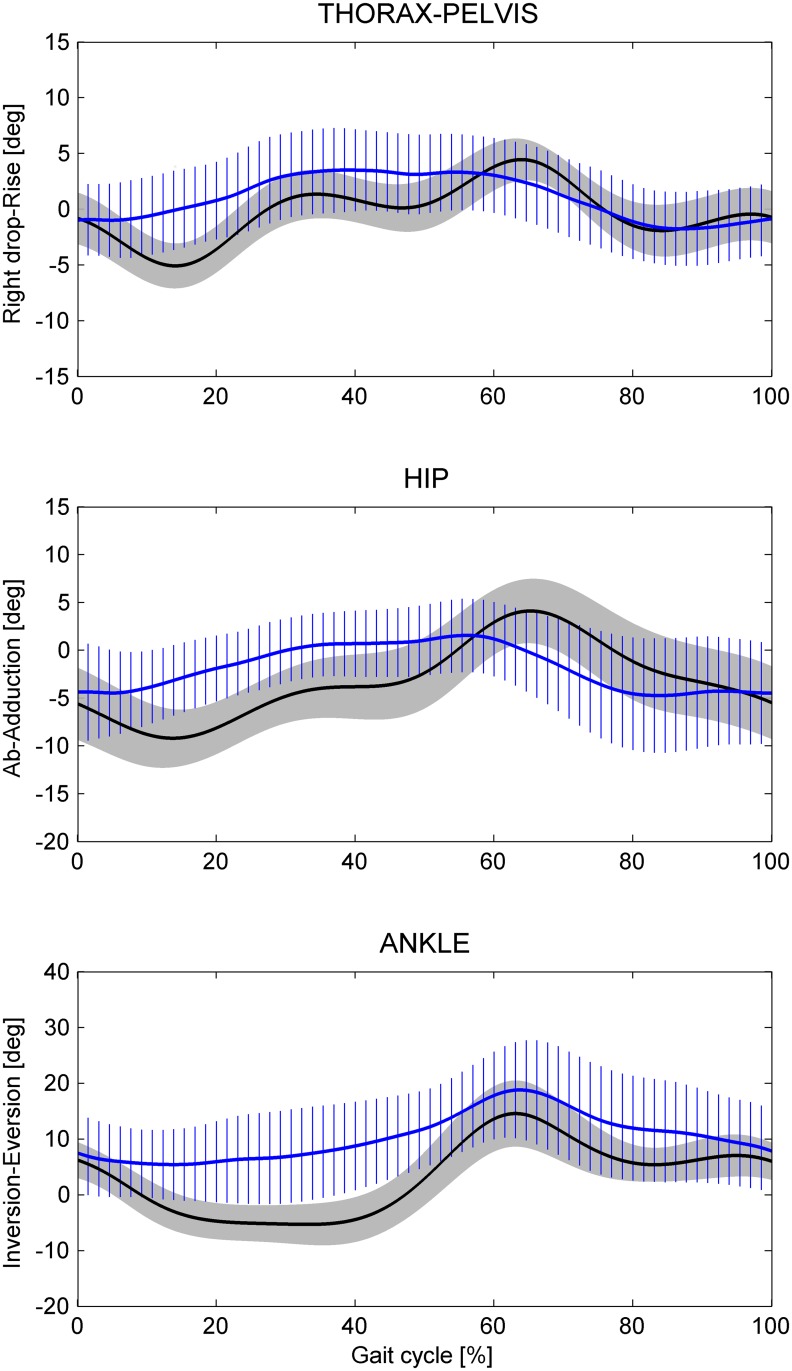
Angular kinematic patterns of the thorax-pelvis, hip, and ankle joints in the frontal plane. Mean values plus and minus one standard deviation for all the participants for Dry-Land (black solid line and grey shaded area) and Under-Water (blue solid line and blue stripes area) conditions.

Globally, regarding the joint angles, similar patterns, but with some differences, were found between the two environments in both sagittal (Fig 2) and frontal (Fig 3) planes. More specifically, looking at the sagittal plane at heel strike, the knee was found to be more flexed (by about 23 degrees comparing the mean values) and the ankle more dorsiflexed (by about 9 degrees) in the UW condition (Table 1, Fig 2) compared to DL. Both these results, can be explained by the different effect of the walking speed in the two environments. A decrease of 0.05 degrees of the knee flexion-extension at heel strike was estimated for each one cm/s increase of the walking speed in the DL condition, while an increase of 0.27 degrees was found in the UW condition. Thus, considering the mean walking speed value, this effect led to a 15.7 degrees of knee flexion at heel strike in UW condition.

Regarding the stance, the peak of the knee flexion that corresponded to the loading response phase typical of the DL was absent in the UW (Fig 2) condition. The hip showed higher values of flexion from the mid stance through most of the swing phase (minimum, toe-off and maximum values as shown in Table 1) under the UW condition. The higher hip flexion at toe-off (about 13 degrees comparing the mean values) can be explained by the different effect of the walking speed in the two environments: a decrease of 0.11 degrees for each one cm/s increase of the walking speed in the DL condition, with respect to an increase of 0.38 degrees in the UW condition. Thus, considering the mean walking speed value, this effect led to a 22 degrees of hip flexion at toe-off in UW condition. In the swing phase, the knee was more flexed also (about 8 degrees comparing the mean values). Moreover, in the sagittal plane the ankle showed a higher range of motion (about 9 degrees comparing the mean values). No differences were found in the mean curve of the flexion-extension angle of the thorax-pelvis joint between the two conditions, except for a higher variability in the UW condition. The higher variability is explained by repeatable but different walking strategies adopted by each single participant in water.

Looking at the frontal plane (Fig 3), the joint angle patterns of the thorax-pelvis and of the hip were smoother in the UW compared to those of the DL condition. In the UW condition, the ankle was more inversed at toe-off (about 7 degrees comparing the mean values) and showed a more inversed mean value (about 7 degrees). These results can be explained by the different effect of the walking speed in the two environments (Table 1). For instance, the ankle inversion at the toe-off showed a decrease of 0.08 degrees for each one cm/s walking speed increase in the DL condition, compared to a decrease of 0.17 degrees in the UW condition.

Only the parameters that showed differences between the two environments were reported in Table 1. The percentage of stance phase and the knee flexion-extension range of motion, even if different from a statistically point of view, were considered comparable from a biomechanical point of view.

Analyzing the sensitivity of (all) the parameters reported in Table 1 to the number of cycles, the maximum difference observed was 1.0 degree for the maximum knee flexion value in the UW condition when comparing the standard deviation obtained with 7 cycles (one trial) and with 32 cycles (three trials) for each subject.

## Discussion

In the present study, thorax-pelvis and lower limb joints kinematics patterns were estimated using waterproofed IMMUs in the UW and the DL walking conditions. Normality bands for young adults were calculated using a high number of gait cycles (352 /704) relevant to the trials performed by 11 healthy subjects. This analysis allowed a comparison between the two conditions, enhancing similarity and differences described more in details in the following paragraphs.

### Spatio-Temporal Parameters

The mean speed of UW walking decreased of 40% with respect to DL walking, consistently with the reduction found in previous studies using video, that varied from 30% [26] to 53% [7]. The stride length was shorter in water (about 10 cm in the mean values) similarly to the stride length reduction of the adults analyzed by Barela [8] using video. Stride lengths and velocities, in both conditions, showed larger values with respect to the literature. Possible explanations of this phenomena are: i) a walking rhythm similar to that in daily life activities, since the use of IMMUs allowed the analysis of several consecutive steps, and ii) taller participants than that of previous studies. The stance phase duration expressed in stride percentage was not modified by the environment like previous video analyses [8,10].

### Sagittal Plane

Comparing the two conditions, the results showed similar patterns in the joint angles with the following differences (Fig 2). During the stance phase and more evidently at the heel strike, a more dorsiflexed ankle and a more flexed knee were observed in UW condition, with mean differences of about 9 and 23 degrees, respectively (Table 1). The hip showed difference during the last phase of the stance, reporting a higher flexion at toe-off (about 13 degrees for the mean values, Table 1). These findings are controversial in literature: some studies [7,12] found mostly the same differences as the results of the present study, while others [8,11] observed substantially no differences at heel strike and toe-off for healthy adults. Probably, these differences among the studies were due to surrounding conditions for example different water depths as a percentage of participants’ heights, different ages, different velocities of walking on level ground at a comfortable speed.

Looking at the linear mixed models analysis, that takes into account singularly and simultaneously the environment and the speed as factors, the differences in the sagittal plane during the stance phase can be explained by the effect of the speed in the water (Table 1). Thus, it seemed that the speed and environment played a major combined role more than any single factor. We might hypothesize that the greater resistance to the movement in water, due to the combination of speed and environment, implies use of a different motor strategy in walking.

The knee flexion peak of the loading response phase typical of the DL condition was not found in water walking, in accord with all previous studies [7,8,11,12]. When the knee joint kinematics was analyzed in the UW condition, extension peak torque in the same phase was not found [11], suggesting the low gravitational weight factor in water as a possible explanation of this phenomenon. However, when bodyweight unloading was investigated in the DL condition [27], the knee flexion peak was observed even up to 75% of bodyweight unloading. Thus, the low gravitational weight might produce smaller changes on knee kinematics than low speed. This hypothesis is supported by previous studies that analyzed the impact of different velocities in DL kinematics of walking [22,27]. Effectively, results did not find the knee joint flexion peak at low velocities [22,27].

During the swing phase, more flexed hip and knee joints were found for UW than for DL condition, with mean differences of about 9 and 8 degrees, respectively (Table 1). Similar results were found in some of the previous studies [9,12], but not in others [8,11], probably due to the different surrounding conditions previously outlined.

The linear mixed models analysis enhanced how these results were explained by the effect of the speed in water (Table 1). The maximum value of knee flexion occurred at the same percentages of gait cycle in both environments, as confirmed by all the studies from the literature [8,11,12]. Probably these results came from the combination of the effects produced by the low speed and the bodyweight unloading. In effect, the first factor produces a time delay [22,27], whereas the second one produces an anticipation [27] of the maximum value of knee flexion in DL walking. The differences in the ankle joint during the swing phase were similar to those found at low velocities [22,27] although they were not significant.

### Frontal Plane

The joint angles patterns of the thorax-pelvis and of the hip in the frontal plane were smoother in UW condition (Fig 3) compared to the DL condition. No comparison could be performed with previous studies that analyzed UW walking since their results were limited to the sagittal plane. However, these findings can be explained by the reduction of the speed, since similar smoother patterns were observed also at low velocities in the DL condition [22]. Interestingly, the ankle showed more inversion values particularly in the stance phase (Fig 3) in the UW condition. Analyses on this angle have not been reported by previous studies, nor in the DL condition at different velocities or in the UW condition.

The sensitivity analysis of the results using different number of cycles showed that the acquisition of one trial using an ICC threshold of 0.75 leads to similar results of those obtained from three trials. However, attention must be paid to the subject familiarization with the water environment and to the algorithm used for the selection of the gait cycles.

### Limitations of the Present Study

The main limitations of the present study can be addressed to the instrument and to the environment. First of all, non-invasive motion analysis instruments, such as IMMUs and optoelectronic systems, are less reliable and showed the highest error in analyzing the joint kinematics of the lower limbs in the transverse plane [21]. For this reason, the present study limited the 3D joint kinematics analysis only to the sagittal and the frontal planes. While optoelectronic systems are widely considered the most accurate non-invasive systems to perform gait analysis in indoor condition, they are not suitable or practical for use in water. Despite a lower accuracy, IMMUs can represent a valid alternative and have several advantages relative to optoelectronic systems, such as wider field of acquisition, faster set-up, and a practical calibration. A limitation is that the IMMUs wireless transmission does not work in UW condition. Thus, the data collected by each IMMU are stored in their internal memories not allowing real time applications. However, for gait analysis it is more important to promptly provide the kinematic patterns at the end of the trials rather than a real time visual feedback.

## Conclusion

The use of the IMMUs, by means of fast set-up and data analysis, allowed an immediate gait analysis just after the execution of the walking task, in both UW and DL conditions. Thus, the methodology adopted can make available a report to the therapist during the aquatic therapy session. In the present study, for the first time, a comparison between DL and UW walking patterns of thorax-pelvis and lower limb joint angles in the sagittal and in the frontal planes was performed using IMMUs. The number of gait cycles acquired was not limited by a restricted field of view as with UW cameras allowing a more robust estimation of the normality bands. The differences with respect to the DL condition enhanced the necessity of further investigations on different water level depths and different walking speeds to better understand how the water environment influences the different motor strategies adopted by the participants.
